# An Evaluation of the Referral Process From General Practice (Gp) to the North-West Community Mental Health Team (Nw Cmht)

**DOI:** 10.1192/bjo.2022.387

**Published:** 2022-06-20

**Authors:** Kathryn Flew, Christiana Elisha-Aboh, Shaharyar Alikhan

**Affiliations:** Leeds and York Partnership NHS Foundation Trust, Leeds, UK, Leeds, United Kingdom

## Abstract

**Aims:**

As more emphasis is placed on a move from the traditional hospital-based practice to care in the community, CMHTs are becoming the main channel for delivering specialist care in England. Access to most CMHTs occurs via written referrals, which vary significantly in content and quality. Such variability and inconsistency with the information provided can impact on the triage process and delay access to treatment for patients, making the process unnecessarily protracted and time consuming. One key factor that would drive the success and survival of CMHTs is how they gate-keep their service. This starts by adopting formal strategies when vetting and screening referrals. The aims were to determine if NW CMHT is responding to referrals appropriately, to consider if service users received good quality correspondence about referral decisions and if the outcomes of such meetings were properly documented.

**Methods:**

The NW CMHT consists of 4 pods (A to D) and the audit included all GP referrals assessed by pod B over a month. A sample size of 28 referrals was included in the audit and the referrals were from 16 different GP practices. Data were obtained from patient electronic records and entered onto a SmartSurvey form for ease of collection prior to results being analysed.

**Results:**

32% of referrals came from two GP surgeries. Areas of good practice included all referrals being discussed within 4 days of receipt, and 50% reviewed by the next day. For referrals identified as needing further information and discussion, this was also done quickly between 2–5 days of receiving the referral. Also 68% of service users (SU) had a letter sent out to them within 2–5 days. It was unclear in 75% of referrals whether the SU was aware of the referral to NW CMHT and the reasons for the referral were only ‘fully’ documented in 57%.

**Conclusion:**

The vast majority of GP referrals were treated in a timely manner, even if additional data gathering was needed and multiple referral discussions had. Recommendations included addressing the lack of consistency in documentation of referral discussions, developing effective ways to cut back on clinical time lost gathering what should be standard information, and education of GP practices around making good quality referrals. It was felt that a review of the referral forms would be beneficial, however a barrier to this change was that this is a trust wide form and there would need to be consensus across all CMHT localities.

